# ‘It's not everybody's snapshot. It's just an insight into that world’: A qualitative study of multiple perspectives towards understanding the mental health experience and addressing stigma in healthcare students through virtual reality

**DOI:** 10.1177/20552076231223801

**Published:** 2024-01-04

**Authors:** Raul Szekely, Oliver Mason, David Frohlich, Elizabeth Barley

**Affiliations:** 1 School of Psychology, 3660 University of Surrey, Guildford, UK; 2 Digital World Research Centre, 3660 University of Surrey, Guildford, UK; 3 School of Health Sciences, 3660 University of Surrey, Guildford, UK

**Keywords:** Virtual reality technologies, mental health stigma, healthcare students, healthcare educators, lived experience, qualitative research

## Abstract

**Objective:**

The resurgence of virtual reality (VR) technologies has led to their increased use in contemporary healthcare education. One promising application is simulating the experiences of individuals with mental health conditions (MHCs) to reduce stigma among future healthcare professionals. This study set out to explore what those impacted by, or involved in, the education of healthcare students think about using VR in this way.

**Methods:**

One individual interview and five focus groups were conducted with healthcare students (*n* = 7), healthcare educators (*n* = 6), and lived experience experts (*n* = 5). Before sharing their perspectives, participants familiarised themselves with VR equipment and immersive materials simulating MHCs. The constant comparative method and thematic analysis were used to analyse the data.

**Results:**

Participants recognised the acceptability and utility of VR for addressing mental health stigma in healthcare students, emphasising the immersive nature of this technology. However, some participants raised concerns about the limited insight VR could provide into the experiences of patients with the same MHCs and its potential emotional impact on users. Participants recommended the incorporation of interactive, realistic environments with a person-centred focus into future VR-based stigma reduction interventions while stressing the importance of providing healthcare students with opportunities for reflection and support.

**Conclusions:**

Healthcare students, healthcare educators, and lived experience experts highlighted both advantages and barriers associated with using VR to understand the experience of patients with MHCs. Furthermore, the recommendations put forward can inform the design, content, and delivery of VR-based stigma reduction interventions in healthcare education.

## Introduction

Over the last two decades, digital technologies have revolutionised the delivery of healthcare education, training, and services.^1–3^ Notably, virtual reality (VR) has experienced a resurgence in recent years, playing a key role in this transformation.^4^ Whereas VR was first developed more than 50 years ago, its definition continues to be a topic of debate, resulting in a lack of consensus in the literature.^5,6^ For example, some authors define VR strictly as a three-dimensional (3D) digitally generated environment that is experienced using head-mounted displays (HMDs), controllers, and even body-tracking sensors.^7,8^ Others propose a much broader interpretation, encompassing both 3D and two-dimensional (2D) digital environments that, besides HMDs, may also be experienced through less immersive interfaces such as large-scale projections, desktops, and mobile devices.^9–11^

Nevertheless, while different authors may use different definitions, there is an acknowledgement within the scholarly discourse that the levels of immersion can vary between VR-based technologies as a function of their technical affordances and the different sensorimotor contingencies enabled by each of these.^12,13^ This has allowed authors to adopt a more precise categorisation and to distinguish between non-immersive, semi-immersive, and immersive VR.^14,15^ Despite structural differences that have contributed to definitional variations across the years, what is and has always been clear is that all VR-based technologies converge towards the same functional objective – to immerse users, either partially or totally, within a digitally generated environment that replicates one or more aspects of the real world, eliciting naturalistic physiological and psychological responses.^14,16,17^

### Simulating the mental health experience and reducing stigma through VR

It is precisely this function of VR to simulate real-world scenarios that have appealed to and found its way into healthcare education and training.^18–20^ Within the realm of mental health, VR is increasingly used as an educational tool to support the development of clinical and non-clinical skills related to the care of patients with mental health conditions (MHCs).^21–25^ More than that, VR-based interventions portraying the experiences of patients with MHCs, including symptom simulations, serious games, 360 videos, and other virtual applications, are suggested to reduce mental health stigma and improve empathy and understanding.^24,26,27^ For example, Zare-Bidaki et al.^28^ showed that a VR simulation of psychotic symptoms decreased medical students’ stigma and improved their empathy towards patients with psychosis. Similarly, Lam et al.^29^ found that VR simulations portraying the experiences of patients with psychosis, anxiety, and depression improved nursing students’ empathy scores and their attitudes towards mental illness. Recently, Rodríguez-Rivas et al.^30^ also demonstrated the effectiveness of an immersive VR game in which the players experience the challenges of someone with a severe MHC in reducing mental health stigma. Similar findings are reported in other areas of research, including dementia,^31,32^ autism,^33^ intellectual disability,^34^ chronic pain,^35^ and inflammatory bowel disease,^36^ indicating the growing recognition of VR as a novel tool for stigma reduction across the health spectrum.

### The problem of mental health stigma in healthcare

The potential of VR to reduce stigmatising attitudes towards patients with MHCs is particularly important since mental health stigma is a significant barrier to healthcare for this group.^37–39^ For instance, a qualitative study with mental health users of an early intervention service and their carers identified the perceived negative reactions from others, as well as the fear of mental health services as potential reasons for delayed help-seeking.^40^ However, experiences of perceived and enacted stigma, including discrimination, are not confined to the context of mental health services alone as they also occur within the realm of physical healthcare. In another qualitative study, mental health service users explained they feel judged, mistrusted, and avoided by healthcare professionals when seeking general emergency care.^41^ There are also reports of healthcare professionals dismissing service users’ physical symptoms, which made them feel they did not receive the care they needed.^42^ These findings are further corroborated by research showing that many healthcare professionals express negative views towards patients with MHCs.^43–46^ In turn, healthcare professionals’ stigmatising attitudes can have a detrimental impact on patient care quality^47,48^ and, critically, contribute to the worryingly high rates of physical morbidity and mortality seen in this patient population.^49^

Stigmatising attitudes towards patients with MHCs are also prevalent in those studying to become healthcare professionals.^45,50,51^ Although education and increased contact with patients may lead to attitudinal improvements in healthcare students, these effects appear to diminish by the final year of their degree.^52–54^ Congruent with concerns from the wider literature regarding the long-term effectiveness of traditional mental health stigma reduction interventions,^55–58^ this observation justifies the need for incorporating novel approaches such as VR in the education of healthcare students in order to tackle stigma more sustainably.

### Perspectives of healthcare students, educators, and lived experience experts

As previously indicated, VR-based technologies and interventions have the potential to reduce mental health stigma among healthcare students by allowing them to step into the shoes of individuals with MHCs and gain an understanding of their condition.^26^ However, little is known about the perspectives of healthcare students as potential users, healthcare educators as potential facilitators, and patients with MHCs as potential end-beneficiaries in relation to VR as a mental health stigma reduction intervention. These perspectives are important not only for understanding the acceptability and utility of VR but also for considering the broader challenges and implications that could influence its successful implementation within healthcare education, with input from key stakeholders early in the process.^59,60^

Although healthcare students generally find the various applications of VR in healthcare education as acceptable, engaging, and user-friendly,^61–63^ their perceptions of using VR to specifically address mental health stigma are yet to be explored. On the one hand, exploring these perceptions can shed light on whether healthcare students recognise mental health stigma as an issue that needs addressing. On the other hand, it allows for the evaluation of students’ perspectives concerning the usefulness of VR in this context, which, in turn, might impact their intention to engage with the technology.^64^

In addition to students, healthcare educators can provide valuable insights beyond the acceptance of the VR technology itself and delve into the practicalities of facilitating VR-based stigma reduction interventions and integrating them into the healthcare education curriculum. For example, resource management, technical challenges, and alignment with relevant learning objectives have been identified as focal points in previous qualitative work with educators.^65,66^

Equally valuable are the views of those living with MHCs (hereafter termed lived experience experts) as they are most likely to notice, and benefit from, the potential positive impact of such interventions through the interactions had with healthcare professionals and the quality of care received. Furthermore, their insights can be instrumental in informing the content of these interventions, ensuring that the experience of living with an MHC is portrayed in an accurate, meaningful, and respectful manner. The involvement of lived experience experts in the design of educational activities and interventions is a central aspect of contemporary healthcare education^67,68^ and has been shown to enhance the authenticity of educational content related to mental health, to promote compassionate, patient-centred care, and to contribute to the destigmatisation of MHCs.^69–72^

### Aims and research questions

In light of the above, the aim of this study is to explore, using a qualitative interpretive description (ID) approach, the perspectives of healthcare students, healthcare educators, and lived experience experts towards the use of VR to understand the experience of patients with MHCs and to address mental health stigma among those studying to become healthcare professionals. In particular, this study seeks to answer the following research questions (RQs):

**RQ1.** What are the perspectives of healthcare students, healthcare educators, and lived experience experts in relation to the advantages and barriers associated with using VR to understand the experience of patients with MHCs?
**RQ2.** What specific recommendations do healthcare students, healthcare educators, and lived experience experts propose to enhance the design, content, and delivery of VR-based interventions aimed at reducing mental health stigma in healthcare education?

## Methods

### Design

A qualitative ID approach was selected to meaningfully explore the different perspectives towards the use of VR to understand the experience of patients with MHCs and to address mental health stigma in healthcare students. Developed by nursing scholars as an alternative to traditional qualitative stances, ID is used to collate and explain the accounts of various stakeholders towards a shared problem and has now become a well-established approach in healthcare education research.^73^ Whereas ID recognises that reality is socially and experientially constructed, its main goal is not to describe the essence of an experience or a phenomenon (as seen in phenomenological approaches) nor to develop explanatory theories of behaviour (as seen in grounded theory approaches) but rather to use the stakeholders’ accounts as a basis for practice change in a particular context, considering both commonalities and variations in views and experiences.^74–76^

### Participants and sampling

As data saturation is not desired in ID studies and there is no objective guide to determine sample size in qualitative research,^77,78^ a recruitment target of 4–6 participants per group (12–18 in total) was set to allow a diversity of views to transpire without neglecting the richness of each individual account. The proposed recruitment target was in line with those of other ID studies published in the field of healthcare education.^79–82^

A purposive sampling strategy was employed to recruit participants for this study. To be eligible, participants were required to be at least 18 years old, to be able to give informed consent and be willing to take part in the research, to have no (self-reported) history of harming themselves or others, and to be able to communicate in English.

Healthcare students (i.e. undergraduate or postgraduate students enrolled in a healthcare-related degree, including but not limited to nursing, midwifery, paramedic science, nutrition and dietetics, and physiotherapy) and healthcare educators (i.e. clinical or non-clinical professionals involved in the design and/or delivery of educational programmes for healthcare students at the university level) were primarily recruited from UK universities via course leaders and administrative staff, as well as through student societies for healthcare students who disseminated the research opportunity via email circulars. Those interested were invited to read the information sheet and the consent form and to complete an online screening form via Qualtrics. Seven healthcare students, of which three (49.2%) were studying mental health nursing, two (28.6%) midwifery, one (14.3%) child nursing, and one (14.3%) adult nursing, and six healthcare educators, of which two (33.3%) with expertise in clinical psychology, two (33.3%) in nutrition and dietetics, one (16.7%) in child nursing, and one (16.7%) in sport science, took part in the study.

Lived experience experts (i.e. individuals with a current or previous mental health diagnosis who have been engaged in healthcare education activities, such as reviewing program/module curriculum, delivering teaching sessions, supporting student examinations, advising on research projects, etc.) were primarily recruited from UK-based mental health research networks and charities and service user advisory groups through representatives who shared the recruitment materials with their members. Five lived experience experts were recruited and took part in the study.

While the research team received additional informal expressions via email, these did not formalise through the completion of the online screening form and, in consequence, records of these numbers were not maintained. All individuals who completed the online screening form were deemed eligible and subsequently invited to select a convenient date and time for participation in the focus group discussions. No further exclusions were made. The demographic characteristics of each participant group are summarised in Table 1.

**Table 1.** Participant characteristics by group.

Characteristics	Healthcare students (*n* = 7)	Healthcare educators (*n* = 6)	Lived experience experts (*n* = 5)
Age, mean (SD)	27.3 (10.2)	36.7 (7.53)	56.8 (17.5)
Gender, *n* (%)			
Female	7 (100%)	5 (83.3%)	3 (60.0%)
Male	–	1 (16.7%)	1 (20.0%)
Other	–	–	1 (20.0%)
Ethnicity, *n* (%)			
Black, Black British, Caribbean or African	–	–	1 (20.0%)
White	7 (100%)	6 (100%)	4 (80.0%)
Highest educational attainment, *n* (%)			
Degree level or above	1 (14.3%)	6 (100%)	5 (100%)
AS, A level or equivalent	6 (85.7%)	–	–
Previous VR experience, n (%)			
Yes	3 (42.9%)	3 (50.0%)	3 (60.0%)
No	4 (57.1%)	3 (50.0%)	2 (40.0%)

### Data collection

Five focus groups were conducted to collect data. Specifically, a focus group was conducted with lived experience experts, followed by two focus groups with three, respectively, four healthcare students. In addition, two focus groups were conducted with three and two healthcare educators, respectively. In the case of the last healthcare educator group, two participants were present as one educator unexpectedly became unavailable for the focus group discussion. Instead, an individual interview was arranged and conducted at a later date to include their insights. Participant categories were purposefully kept separate to allow for an exploration of similar experiences and expertise within each group, as well as to ensure a balanced power dynamic among participants.

In line with the recommendations of Kallio et al.,^83^ a topic guide was developed based on the available literature, as well as with input from the research team. The topic guide was informed by the Consolidated Framework for Implementation Research^84^ and can be found in Table 2. The topic guide was refined following initial focus group discussions to improve clarity through minor adjustments in question-wording and the addition of prompts.

**Table 2.** Topic guide.

Domain	Questions and prompts
Knowledge and beliefs	In general, what are your thoughts on VR interventions that simulate the experiences of someone with an MHC? For example, an intervention whereby users hear intrusive, distressing voices.
Needs and resources	Reflecting on your experience, do you feel there is a need for delivering VR interventions tackling mental health stigma among healthcare students? Why/why not?
Evidence strength and quality	Do you believe a VR intervention will or can change the way healthcare students view and act towards patients with MHCs? Why/why not?
Adaptability	What do you think VR interventions should include to improve healthcare students’ attitudes and behaviours towards patients with MHCs? What is important to consider? Think about the design process, content, delivery, etc.
Relative advantage	Do you see any advantages or benefits for VR interventions compared to other stigma reduction interventions? Other interventions may be watching an educational video about mental health or interacting with someone with an MHC.
Complexity	In your opinion, what are some of the barriers or challenges associated with the implementation of VR interventions in the education of healthcare students? You may consider ethical aspects, costs, acceptability and uptake, etc.

VR: virtual reality; MHC: mental health condition.

Data collection took place in-person, at a university campus, between May and June 2023. The reason behind the choice of in-person was to enable participants, particularly those with little to no prior exposure to VR, to gain hands-on experience with the immersive VR equipment. The equipment consisted of the Oculus Quest All-in-one VR Gaming Headset, a portable and standalone device offering six degrees of freedom and inside–out tracking through four built-in cameras, along with the second-generation Oculus Touch controllers. Furthermore, the in-person data collection allowed all participants to explore the same illustrative VR materials and understand how VR could be used to portray or simulate the experiences of individuals living with MHCs. These materials were online-sourced, presented in VR or 360 format, and with a relatively short duration (up to 5–10 min), serving as illustrative examples for participants to develop a general understanding of the potential uses of VR in this context and inform their perspectives ahead of the focus group discussions. Examples included ‘Inside Anxiety’ by BBC Scotland^85^ and ‘What Bipolar Disorder Feels Like’ by WebMD.^86^

Data collection was facilitated by the lead researcher who, at the time of conducting this study, was a PhD student with a background in psychology and experience in health-related research. Before each focus group, participants were also informed about the background and purpose of the study, confidentiality, and ground rules, then they were asked to complete a brief demographic questionnaire (e.g. age, gender, ethnicity, education, previous experience with VR, etc.). The focus groups commenced shortly after the VR demonstration. With participants’ consent, all focus groups were audio-recorded using a digital voice recorder (Philips VoiceTracer 4110) for transcription and analysis purposes. The duration of each focus group ranged from 60 to 75 min, allowing sufficient time for in-depth discussions and follow-up questions or further clarifications.

### Data analysis

The constant comparative method, an iterative approach often applied in ID studies,^87–89^ and thematic analysis^90^ were used to analyse the qualitative data. The audio recordings were transcribed verbatim by the lead researcher. Names and any other identifying information were removed to ensure anonymity. The anonymised transcripts were then entered into a qualitative analysis software (NVivo 1.7.1) to support the analysis process. The data analysis process, as well as the interpretation of findings, was discussed and agreed upon by the research team.

The data analysis was conducted by the lead researcher and followed several steps. Immersion into the data co-occurred with each focus group and continued beyond data collection through the iterative reading of transcripts, leading to familiarisation with the qualitative data. Initial remarks underpinning potential patterns were noted and open coding was employed, with descriptive labels assigned to the data. This process resulted in the generation of an initial set of lower-level codes, capturing the explicit content of the data.

Following open coding, axial coding was applied iteratively to identify relationships between the lower-level codes. Axial coding led to the aggregation and condensation of codes into broader, higher-level categories (sub-themes). As the analysis continued, these categories were refined in response to emerging insights, highlighting the iterative nature of the analytical process. For instance, codes such as ‘can help identify problems’, ‘improve relations with patients’, ‘learning to recognise behaviours’, ‘makes you more confident’, and ‘you can’t see all conditions’ all referred to content related to how VR could effectively prepare students for looking after patients with MHCs in a clinical context. Through constant comparisons, these codes converged into a higher-level category labelled ‘Preparation for clinical work’.

The higher-level categories were subsequently grouped thematically into overarching categories, which served as thematic frameworks for structuring and presenting the findings, as they derived directly from the RQs. In presenting the findings, representative quotes were selected based on their richness and relevance to capture the core aspects of each overarching category. Additionally, the overarching categories were compared and contrasted between the different participant groups, shedding light on potential variations in perspectives. While the perspectives expressed were largely similar, if a certain position was endorsed more strongly by a participant or group of participants, this was made explicit in the presentation of findings, providing therefore a more nuanced understanding of the data.

### Trustworthiness and rigour

To maintain the rigour and trustworthiness of the research process, a number of strategies were adopted. Firstly, participants were invited to review the transcripts of their own focus group discussions to verify the accuracy of their accounts and ensure that their views, experiences, and opinions were accurately represented in the analysis. Then, the analytical plan was defined a priori in the study protocol and jointly agreed upon by the research team, which consisted of experts in clinical psychology, interaction design and digital media, and nursing. In addition, any challenges during the analysis process were discussed with members of the research team as often as possible and differences in interpretation were resolved through consensus. An audit trail was also kept for recording any changes to coding and analysis. Finally, the Standards for Reporting Qualitative Research^91^ were used to inform the reporting of this study.

### Ethical considerations

This study received a favourable ethical opinion from the University of Surrey Ethics Committee (FHMS 22-23 126 EGA) and was carried out in accordance with the principles of the British Psychological Society Code of Human Research Ethics.^92^ Participants were fully informed about the purpose of the study and assured of the voluntary nature of participation and their right to withdraw. Participants provided written informed consent electronically, via Qualtrics, at the time of signing up for the study, prior to data collection. Additionally, verbal re-confirmation of consent was obtained from participants before each focus group. Sources of mental health support were indicated in the information sheet and reminded during data collection. Transcripts were anonymised to protect identities and participants were assigned unique study numbers (1–18) by their roles (lived experience expert, healthcare educator, and healthcare students). £20 Amazon e-vouchers were offered to thank participants for their time.

## Results

**Figure 1.**
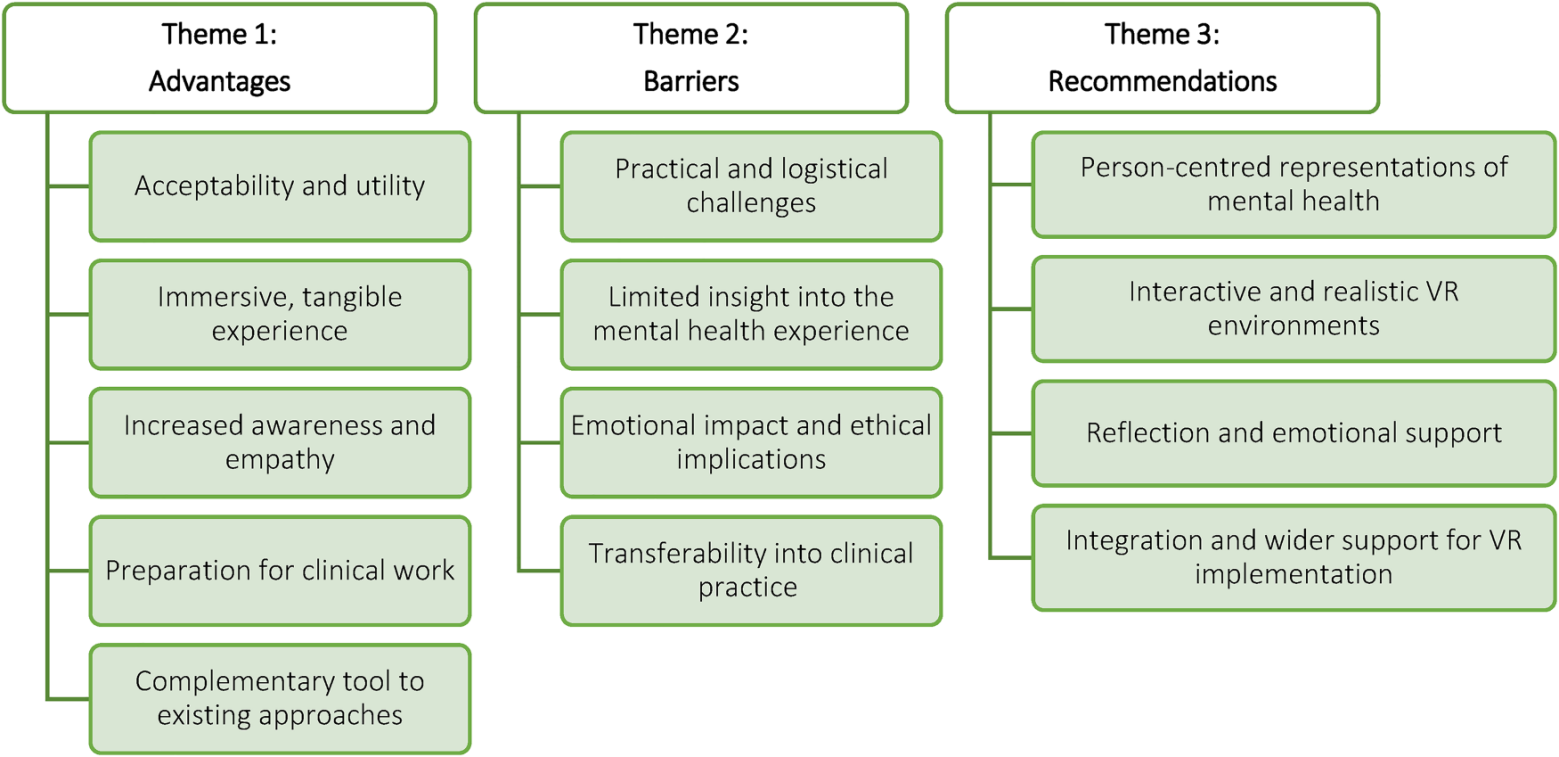
Diagram illustrating the constructed themes and corresponding sub-themes.

Three themes and 13 sub-themes were constructed as a result of the data analysis process (see Figure 1). These themes highlight (1) advantages and (2) barriers associated with the use of VR to understand the experience of patients with MHCs and to address mental health stigma in healthcare students, and (3) recommendations in relation to the design, content, and delivery of VR-based stigma reduction interventions for healthcare students in order to maximise their impact.

### Theme 1: advantages

#### Acceptability and utility

Participants discussed the various applications of VR in healthcare education and found it to be an acceptable and useful tool for teaching healthcare students about mental health stigma and the experience of patients with MHCs. For example, participant 17, who was a healthcare educator, said:

I think it's probably gonna have its place within, certainly as an educator and educating nurses of the future and healthcare professionals, it's definitely gonna have its place. (P_17_, healthcare educator)

They characterised the VR as ‘engaging’ (P_13_, healthcare educator), ‘memorable’ (P_15_, healthcare educator), and ‘interesting’ (P_2_, lived experience expert), aspects which they believed would make healthcare students eager to try this technology. According to some participants, younger students would be more open to it as they are generally ‘more attuned with technology’ (P_7_, healthcare student). Others thought that healthcare educators and practitioners would equally embrace the use of VR due to its novelty element.

It's new, so everybody is starting from scratch with this and so they’ll come on board because it's new. It's a new device. VR. Wow. I wish we’d had this when I was training, they may even say. (P_4_, lived experience expert)

Although healthcare educators and lived experience experts acknowledged the growing attention on mental health in healthcare education, healthcare students, particularly those on general healthcare courses, expressed a sense of inadequacy in their preparedness to effectively care for patients with MHCs. Students also acknowledged the link between physical and mental health and recognised the value of VR in increasing their understanding of what a patient with an MHC might experience.

There was a paramedic and an adults nurse and yesterday we were talking about mental health and they were saying that they really haven’t had that many lectures on mental health at all. And the paramedic in particular was saying like ninety percent of the people that come to us have a mental health issue or something related to it. […] And even having something like the headset just to be like ok, we’re going to somebody who has got suicidal ideation and it has psychosis or whatever. Just having that understanding. It will only take 5 min or so just to have an understanding. (P_9_, healthcare student)

#### Immersive, tangible experience

Participants described the immersive nature of the VR experiences and the impact it had on them, from the

bombardment of different sensory experiences (P_1_, lived experience expert)

and

‘hearing the cognitions of the person’ (P16, healthcare educator) to the ‘bodily sensations […] like the heart racing, as well as the flashing of lights’. (P3, lived experience expert)

Participant 1, a lived experience expert, explained the following:

Just the visceral kind of impact of it I think is, that's what I think people are after here, is the visceral impact of appreciating a particular experience or condition, mental condition. (P_1_, lived experience expert)

Participants also described the VR experience as ‘intimate’ (P_14_, healthcare educator), ‘inescapable’ (P_9_, healthcare student), and feeling ‘enclosed into the scenario’ (P_11_, healthcare student). Few healthcare students found the experience uncomfortable but realised that these encounters mirrored the challenges faced by individuals living with MHCs.

It’s a little, it’s a little bit distressing, but so is what they’re experiencing, you know. It’s kind of something a little bit necessary to be like, oh, ok, like, this is really real to you. And I can now relate to you, considering I’ve never experienced that before. (P_9_, healthcare student)

Nevertheless, participants felt that VR could help ‘bring to life’ (P_2_, lived experience expert) and allow them to visualise an experience that would otherwise be ‘very hard to imagine’ (P_14_, healthcare educator). Even though they were aware of the heterogeneity of mental health experiences, symptoms and conditions, participants recognised that using VR in this way would approximate the actual lived experience as closely as possible.

#### Increased awareness and empathy

Participants emphasised the potential of VR in increasing healthcare students’ awareness of mental health and its associated challenges. They highlighted how VR could serve as a powerful tool to increase awareness, ultimately contributing to the reduction of stigma towards patients with MHCs.

I think a key part of kind of stigma formation is knowledge. And so I think this is where that kind of comes in. It's that myth busting almost of, kind of what is it like to have these experiences and if you can increase people's like mental health literacy, then that would hopefully have some kind of knock-off effect. (P_18_, healthcare educator)

Lived experience experts also mentioned the role of VR in cultivating empathy among healthcare students by enabling them to step into the shoes of individuals with MHCs. By doing so, healthcare students could gain an emotional appreciation of the individual's journey and their condition, as participants highlighted during the discussions.

And you’re hearing as if it's in your head and you’re experiencing now, a young student, whoever is sitting in the chair […] and now they’re understanding, oh my god, this is awful for this person. And then you’ll know exactly what it's like, you as a student, how it is and how uncomfortable it is there when you’re suffering from anxiety, whatever mental illness is. And then it's an eye opener for them as it would be for a consultant to really know the little forensic details of how it is when you’re about to go into a panic attack. (P_4_, lived experience expert)

#### Preparation for clinical work

Participants agreed that VR could provide a safe and controlled environment for healthcare students to engage in experiential learning and set realistic expectations concerning the challenges they might encounter when entering clinical work. Healthcare educators also believed that VR would enhance healthcare students’ confidence and alleviate their anxiety as they transition into clinical work, empowering them to approach real-world clinical scenarios with a greater sense of readiness and self-assurance.

So I think if they’d had this kind of thing, would be like, actually, these are the kind of things you’re actually going to see. Like yes, you might see someone hitting their head against the wall, but they’re not actually gonna be harming you […] They would then be better prepared to actually do that and go on to mental health placement in real life. So it as an advantage to me as a clinical educator, it means that they’ve got a bit of a head start in terms of preparation to get them ready. So they’re not as scared, for want of a better word, to go into that environment because they’ve seen the kind of things that can happen. (P_15_, healthcare educator)

Moreover, students discussed how VR could positively impact the development of clinical and communication skills when caring for patients with MHCs. They expressed that VR could help healthcare students to better recognise signs and symptoms, understand their patients’ experiences, and provide appropriate care.

I think it would help us often in practice. Say I worked with a midwife and she had a lady who committed suicide after she had her baby and the midwife is a lot more on it with mental health because of their experience. And I think like using a VR headset would mean that we were more aware, more on it in terms of like asking the questions and just looking out for the symptoms. (P_11_, healthcare student)

#### Complementary tool to existing approaches

Many participants, including the lived experience experts, felt that VR provided a richer and more engaging learning experience compared with more traditional approaches such as simulations and role plays, educational videos, and lectures. However, educators and students, in particular, noted that VR should be viewed as an additional tool rather than a substitute, serving as a complement to existing approaches in healthcare education.

I think maybe it's not a replacement of. I think maybe this, I think there's a place for each intervention […] It might be after you’ve used all of those interventions, you might think actually this one's better, which is your immersive experience. So I don’t think it's necessarily isolating the others, you know, shelving them in favour of. I think maybe it's learning from the pros and cons of each and kind of building something that is possibly a more go-to option. (P_7_, healthcare student)

Additionally, participants appreciated that VR could cater to different learning needs and preferences, as ‘some students respond really well to … visual’ (P_15_, healthcare educator), while others prefer hands-on experiences. They underlined that VR would tap into ‘another form of learning’ (P_15_, healthcare educator), and could engage a wider range of healthcare students. Overall, participants thought that VR would be a valuable addition to the educational toolkit, with the potential to create ‘breakthrough’ (P_4_, lived experience expert), moments in healthcare education and practice, especially if combined with other approaches.

### Theme 2: Barriers

#### Practical and logistical challenges

Participants raised several practical and logistical challenges associated with the use of VR in healthcare education. They believed the cost of VR equipment and maintenance could be significant, posing a financial obstacle for universities. Participants were also concerned about the potential equipment breakages and the costs of repairs and replacements, drawing attention to the financial implications of addressing such issues.

Furthermore, healthcare educators explored the possibility of incorporating VR into larger classes, highlighting the challenges they anticipated, such as the management of multiple VR sets, the need for adequate technical training, as well as the need to allocate sufficient time for reflection and discussions with students.

For me, from a practical perspective is, if you’re gonna do this with students like how many sets you got, how long is it gonna take? If you have a class of 30, how are you gonna manage that and then have time to reflect on afterwards? So that would be an issue for me really. (P_13_, healthcare educator)

Participants also discussed the contraindications associated with VR, particularly in relation to specific health conditions such as epilepsy and balance disorders. They recognised that VR might exclude individuals with these conditions, potentially affecting a proportion of healthcare students who would be unable to use VR as part of their education.

#### Limited insight into the mental health experience

Participants across all groups voiced concerns about the limitations of VR in providing a complete understanding of mental health. They acknowledged that capturing the full breadth of mental health experiences would be challenging as individuals with the same diagnosis can have vastly different presentations and lived experiences. Participants generally viewed VR as a valuable snapshot or insight into the mental health experience but recognised that no single representation can fully capture everyone's experience:

It's, it's a snapshot. It's not everybody's snapshot. It's just an insight into that world. (P_7_, healthcare student).

Participants also drew attention to the risk of VR experiences shifting the emphasis to symptoms, potentially overshadowing the individual's unique journey and failing to appreciate the nuanced and complex nature of MHCs. Critically, lived experience experts expressed concerns that the limited insight provided by VR could result in healthcare students having a false understanding of mental health, leading to potential oversimplification and generalisation when interacting with real patients.

So the virtual reality puts a great big kind of huge emphasis on a particular mental model. And then people might mistake that for this is real, I have a real understanding now. I can go and, and act on that understanding. And, of course, the actuality will not resemble the model and the extent to which it departs will be the extent of any kind of clash or error in how the practitioner responds to individuals. (P_1_, lived experience expert)

#### Emotional impact and ethical implications

Another barrier identified by participants was the emotional impact that a VR experience might have on healthcare students and the potential ethical implications of using VR in healthcare education, including the need for careful planning, support, and trigger warnings. Many healthcare educators stressed that VR experiences, with their immersive nature, have the potential to evoke strong responses and could be distressing for some students, especially those who have personal lived experiences related to the content being depicted.

We have to be so careful with how we go about that with the students that we’re educating and their, the experiences they already have. Some of them, many of them already have had their own personal mental health experiences and potentially we run the risk of, we kind of run the risk of potentially inducing things or, or bringing back memories of things they’ve experienced. (P_17_, healthcare educator)

In contrast, some healthcare educators argued that healthcare students should be exposed to potentially triggering situations in VR to better prepare them for real-world clinical practice, where trigger warnings are not available. They believed that experiencing challenging scenarios in a controlled VR environment could help healthcare students develop resilience and learn to manage their emotional responses. However, it was recognised that debriefing sessions and ongoing support are necessary to help students process their experiences and protect their emotional well-being throughout their education.

#### Transferability into clinical practice

Lived experience experts shared their concerns about the potential challenge of transferring learning from VR environments to clinical practice. They acknowledged that whereas VR can provide valuable learning experiences, there is uncertainty about whether the understanding gained through VR would translate into compassionate and empathetic behaviours towards patients with MHCs when professionals are confronted with the realities and pressures of the clinical setting such as workload and time constraints. For instance, Participant 1 stated:

The biggest challenge is the work environment. […] The idea that, that you can have an attitude while you’re in a learning environment, but when you’re in an operational environment, you can be overwhelmed by the difficulties that are presented in that environment. (P_1_, lived experience expert)

In addition, both healthcare students and lived experience experts mentioned the potential reluctance of experienced healthcare professionals towards new practices. However, they expressed optimism that the fresh perspectives and understanding of today's students have the potential to challenge established norms and contribute to reducing the stigma. As Participant 9, a healthcare student, shared:

The students that we’d use [VR] on now would become the professionals in years to come. So I think that involving students in university can only help the NHS down the line. (P_9_, healthcare student).

Altogether, these perspectives reflect the belief that the engagement of healthcare students in VR-based stigma reduction interventions could bring positive changes and influence the attitudes and behaviours of more experienced professionals in the future clinical setting.

### Theme 3: Recommendations

Healthcare students, healthcare educators, and lived experience experts provided several recommendations regarding the design, content, and delivery of VR-based stigma reduction interventions in healthcare education. These recommendations, which were grouped thematically and summarised in Table 3, reflect the participants’ insights into how the impact of VR interventions can be maximised, drawing upon the identified advantages and barriers.

**Table 3.** Participants’ recommendations for maximising the impact of VR-based stigma reduction interventions.

Sub-themes	Recommendations	Illustrative quotes
1. Person-centred representations of mental health	Promote diversity and inclusion: VR content should include a range of characters reflecting various demographics (e.g. age, race and ethnicity, sex and gender, etc.), promoting inclusivity, and providing a sociocultural perspective on mental health experiences.	‘And then whoever's making these [VR simulations] can then introduce more, lots of permutations. And go to the areas where things are still lacking. Maybe somebody having a psychotic episode in the street […] And diversity as well, the backgrounds, [the VR character] could be a Black person, they may be white.’ (P_4_, lived experience expert)
	Address the complexity of mental health: Move beyond a symptom-focused approach and explore different aspects of the mental health experience through a person-centred lens. Expand the VR content to capture a wider range of experiences and narratives (e.g. discrimination, recovery, etc.), including the intersection with other conditions.	‘[…] getting something that reflects lived experience, but that lived experience looks very different. Even people with the same diagnosis have very different presentations and symptoms and lived experience. So how do you capture that diversity into a single programme?’ (P_18_, healthcare educator)
	Collaborate with service users and lived experience experts: Involve individuals with lived experience of MHCs in the design of VR content to provide authentic insights and ensure the content resonates with their experiences.	‘Getting it right for each illness. That’ll be a challenge. That's why I think you should talk to a service user group before you make them or before or show them what's already made. And see what they think.’ (P_2_, lived experience expert)
2. Interactive and realistic VR environments	Design clinical-like environments: VR environment should closely resemble the clinical setting, including realistic visuals, sounds, and other environmental features that create a high level of realism.	‘You could teach people to work in the NHS with all of the aspects of being in the NHS, like if you’re in the NHS, the room doesn’t look like this […] It's too hot because they’ve got this old heating system and the lights are flickering and there's a phone going as someone comes in, they booked the room as well, and all kinds of stuff.’ (P_16_, healthcare educator)
	Incorporate interactive elements: VR interventions should allow healthcare students to actively respond to challenging situations, make decisions, and interact with other characters (e.g. patients, healthcare professionals, etc.).	‘I think it would be useful for us as well though if you had like the VR, it was like a simulated scenario and it was a healthcare professional managing the situation. Because a lot of the time you don’t see it, you’re not put with the staff member that's managing it. You haven’t really got any idea on how to actually deescalate the situation.’ (P_11_, healthcare student)
3. Reflection and emotional support	Facilitate reflective discussions: Encourage structured discussions among healthcare students following VR intervention, allowing them to share their thoughts, emotions, and learnings related to the experience.	‘So they need to have the, the intervention, but then have the opportunity to kind of talk about, OK, what does this mean for my client? What does this mean for me? How do I interact with that? Cause otherwise they’re not going kind of use the learning or use the experience that they’ve had.’ (P_15_, healthcare educator)
	Provide emotional support: Offer support mechanisms following VR intervention to address the potential emotional impact on healthcare students. These may include debriefing sessions, self-care resources, and signposting to mental health and well-being services.	‘I think it's important for it to be brought in, in a very supportive framework. Not just showing the VR and then that's it. And with advice about, a debrief time as I said before, and self-care advice and guidance and support.’ (P_5_, lived experience expert)
4. Integration and wider support for VR implementation	Ensure meaningful integration into the curriculum: Integrate VR into the healthcare education curriculum in a meaningful, thought-out manner, while considering the diverse needs of healthcare students and ensuring accessibility and flexibility in the use of VR-based resources.	‘And just I think making sure that it's done not just for the sake of it, but doing it actually with a purpose and and, you know genuinely fitting within the curriculum rather than just, you know, we’ve got this great new bit of tech, let's bring it in it. It's got to have, it's got to be purposeful and meaningful’ (P_17_, healthcare educator)
	Prepare the educators: Provide training, support and resources for healthcare educators to equip them with the necessary skills and knowledge to effectively use VR-based technologies.	‘I think there would definitely need to be, you know, I guess, staff training as well around actually, how are we gonna put that in and actually, how are we gonna scaffold around that with supporting the students, preparing the students and debriefing the students and doing it in a a safe way.’ (P_17_, healthcare educator)
	Gain buy-in from stakeholders: Engage with key stakeholders, such as university leaders, funding bodies, ethics committees, industry partners, etc., and demonstrate the value and impact of VR in healthcare education to gain buy-in, secure funding/resources, and facilitate the implementation at a larger scale.	‘That has to be somehow passed, so ethics or whoever else needs to look at it […] But if the learning worth is big enough and huge enough, then they will look at it and then it’ll be up to you or whoever's making this to sort of sell that to them and say this is really powerful and you need to watch it.’ (P_4_, lived experience expert)

VR: virtual reality; MHC: mental health condition.

## Discussion

The healthcare students, as well as the healthcare educators and the lived experience experts taking part in this study, perceived VR to be an acceptable and useful tool in providing an understanding of the experience(s) of patients with MHCs. This finding is congruent with results from previous research investigating the acceptability of patient-embodied VR in healthcare education.^31,62^ For example, nursing students found a VR simulation reflecting the clinical symptoms of schizophrenia to be easy to use but also useful for the development of clinical and communication skills.^62^ Similarly, medical and pharmacy students taking part in a VR dementia experience acknowledged its usefulness in increasing their understanding of the condition but also in motivating them to provide better care to patients with dementia.^31^

Nevertheless, while acceptability and utility are important preconditions for the uptake of technologies such as VR,^64,93^ our findings indicate that practical and logistical factors are essential to consider in an applied context, outside the experimental lab. Consistent with past research,^65,66,94^ financial costs for universities, technical challenges in the set-up of VR equipment, and the management of the learning activity were discussed as potential factors that could hinder the implementation of VR within healthcare education. In response to these issues, gaining buy-in from key stakeholders, providing technical training and support to educators, and facilitating the meaningful integration of VR within the healthcare education curriculum were put forward as recommendations. These recommendations are aligned with the conclusions of a recent scoping review by Lie et al.,^60^ suggesting that, in order to be successful, the implementation of VR in healthcare education should be a well-orchestrated and collaborative effort between all stakeholders. However, it is important to acknowledge that the implementation of VR requires monetary resources, and, despite the enthusiasm for the novelty element and the growing body of evidence, there is a scarcity of reports on the cost implications of such interventions which are needed to fully understand their financial impact and sustainability within the existing healthcare and educational landscape.^95,96^

Furthermore, the present study shows there is public support for the use of VR as a mental health stigma reduction intervention for healthcare students. This is particularly important because many healthcare professionals and students express stigmatising attitudes towards patients with MHCs.^45,50,51^ Meanwhile, there continues to be a suboptimal provision of mental health education and training within general health specialities,^97–99^ problems that were also raised by participants in our qualitative research. In this context, participants viewed VR as a tool that has the potential to effectively increase awareness and cultivate empathy towards mental health.

A notable advantage of VR is the ability to transport users into the world of individuals living with MHCs and to enable them to adopt their perspectives.^100,101^ In our study, and similar to other qualitative research,^102^ this feature was particularly appreciated due to the sense of immersion and the authentic psychophysiological reactions enabled by the use of HMDs. This immersive experience allowed participants to gain a deeper understanding of how living with an MHC might impact a patient's life, something that would be difficult to grasp or imagine otherwise. Interestingly, while some participants found the immersive experience to be slightly unsettling, it was generally acknowledged that this emotional response would be integral to reducing stigma. The potential emotional impact on students was also a matter of concern, and, as a result, it was recommended that adequate support should be in place, including access to counselling services or debriefing sessions to address any negative emotional reactions that may arise. The emphasis on the emotional impact highlights the importance of a balanced approach to implementing VR-based interventions into healthcare education, especially when the goal is to address and reduce mental health stigma rather than unintentionally amplify it.

At the same time, a notable barrier was the limited insight provided by VR into the full breadth of mental health experiences, given the variability of presentations and experiences across different MHCs but also within the same MHC.^103–105^ It was recognised that while valuable, this insight may not fully reflect the multifaceted nature of MHCs and largely focuses on symptoms. This led to concerns about potential oversimplification and generalisation by healthcare students when interacting with real patients, potentially perpetuating stigma. To address this limitation, participants recommended the development of person-centred (as opposed to symptom-focused) representations of mental health within VR content. These representations should prioritise diversity in terms of demographics and experiences, including the complex intersections of mental health with other aspects of life. Collaboration with service users and lived experience experts also emerged as a crucial step to ensure that VR content authentically captures this complexity.

Nonetheless, beyond its potential for addressing mental health stigma, integrating VR into healthcare education is perceived as a valuable complementary tool to more traditional approaches (e.g., simulations, role plays, educational videos, lectures, etc.). VR can help healthcare students develop their confidence and skills in a controlled and safe environment before going into clinical practice and interacting with patients.^106,107^ However, the transferability of VR-based learning to real-world clinical settings can be problematic, an issue often raised in the simulation literature.^108,109^ According to participants in our study, the VR environments should contain realistic aspects of the clinical setting, as well as interactive elements so that healthcare students can learn how to act as clinicians in high-pressure scenarios. This approach is expected not only to enhance students’ understanding of MHCs from a first-person perspective but also to equip them with the necessary skills to effectively care for patients with MHCs in a real-world setting, bridging the gap between VR and the actual clinical reality.^110^

### Implications for research and practice

To the best of our knowledge, no studies have previously explored the perspectives of healthcare students, healthcare educators, and lived experience experts towards the use of VR to understand the experience of patients with MHCs and to address mental health stigma among those studying to become healthcare professionals. This study fills therefore a critical gap in the literature and provides a starting point for future research and practice in the realm of mental health stigma reduction in healthcare education through the use of VR-based technologies.

In light of the current findings, VR emerges as a valuable tool to address mental health stigma among healthcare students by providing them with a first-hand insight into the experiences of individuals with MHCs and increasing their understanding before going into clinical practice. However, for VR to become an effective part of healthcare education, a broader integration strategy is essential. Universities and healthcare educators should prioritise the meaningful incorporation of VR into the existing curriculum. To maximise its impact, healthcare educators should also receive adequate training in the use of VR, ensuring that it aligns with educational objectives and remains sensitive to the needs of students. Additionally, healthcare students who engage with VR content should be provided with appropriate support systems to process any emotional reactions and reinforce their understanding of MHCs.

Further, it is important for researchers to recognise the need for collaboration in the development of VR-based stigma reduction interventions. For example, lived experience experts can bring invaluable insights into the authentic portrayal of mental health experiences, ensuring that the VR content is accurate, meaningful, and respectful, whereas healthcare students and educators can ensure that the scenarios address relevant learning needs and maintain a good level of clinical realism. In this way, researchers can co-create interventions that not only enhance understanding of the experiences of patients with MHCs and reduce mental health stigma but also better prepare healthcare students to be sensitive to the needs of this patient group, ultimately improving the quality of care they provide as future professionals.

### Strengths and limitations of the qualitative study

There are a number of strengths to this qualitative study. Firstly, the inclusion of three distinct participant groups – healthcare students, healthcare educators, and lived experience experts – ensured the exploration of relevant perspectives within the context of using VR as a mental health stigma reduction intervention in healthcare education. This allowed for the recognition of both commonalities and variations in perspectives across groups, as well as within each group, given participants’ unique experiences, beliefs and opinions, therefore fostering a critical and balanced discussion. Secondly, another strength refers to the VR demonstration which offered participants, especially those new to VR, the opportunity to become familiar with the technology and gain a practical understanding of how it can be applied in the context of mental health stigma reduction. This, in turn, ensured that their contributions to the focus group discussions were as informed as possible. A final strength lies in the use of a methodologically flexible approach such as ID. This approach facilitated the exploration of perspectives towards an applied problem, whereby the focus is on shaping practical applications and informing practice change rather than describing the essence of an experience or developing theories of behaviour.

This study also presents some limitations. It is important to acknowledge that participants exclusively engaged with an immersive technology, namely the HMD, during the VR demonstration. In consequence, all discussions and findings in this study predominantly refer to this immersive VR technology. It remains less clear how participants might perceive other VR-based technologies in relation to their potential to simulate the experience of patients with MHCs and address mental health stigma.

Exposure to specific VR content, as well as the overall technical interaction with the immersive VR equipment, could also have influenced participants’ perspectives. To prevent potential issues, all participants received guidance from the lead researcher on how to use the equipment for an optimal experience. In addition, following the VR demonstration, and before the commencement of the focus group discussions, participants were given the opportunity to reflect on their initial impressions. However, it was important that participants shared their thoughts and perspectives about whether and how (immersive) VR in general could be used to address mental health stigma. Therefore, during the discussion, they were reminded not to focus on the specifics of the materials they had viewed as these served as illustrative examples only.

Finally, the majority of participants were from the same geographical area in the southeast of England which has a predominantly White population. It is important to note, however, that data collection was conducted in person, which might explain why participants living closer to the study location were more likely to take part as opposed to those from more distant geographical areas. At the same time, while the ethnic makeup of the sample can potentially be attributed to the geographical distribution, the predominance of female participants, particularly within the healthcare student group, aligns with the typical gender distribution observed among students enrolled in healthcare courses in the UK.^111,112^

Nevertheless, due to the limited demographic diversity of the sample, the findings might not necessarily reflect the perspectives of other groups which, for various reasons (e.g. cultural norms, socioeconomic disparities, etc.), might have different attitudes towards mental health stigma and technology use. While this consideration is significant, it is equally essential to acknowledge that, by definition, ID studies are focused on providing a nuanced understanding of a problem or phenomenon within a particular context, rather than aiming for generalisable findings.

## Conclusions

VR-based technologies have regained momentum decades after their initial development and are being increasingly used in many aspects of contemporary healthcare education. A novel application is provided by the potential of VR to simulate the experiences of patients with MHCs, which can also act as a stigma reduction intervention for those preparing to become healthcare professionals. This qualitative study set out to explore what those impacted by, or involved in, the education of healthcare students think about using VR in this way.

Participants recognised the acceptability and utility of VR for addressing mental health stigma in healthcare students, emphasising the immersive nature of this technology which could complement more traditional approaches. However, there were concerns about the limited insight VR could provide into the experiences of patients with the same MHCs and its potential emotional impact on users, as well as practical and logistical challenges pertaining to its implementation in healthcare education. Participants recommended the incorporation of interactive, realistic environments with a person-centred focus into future VR-based stigma reduction interventions while stressing the importance of providing healthcare students with opportunities for reflection and support.

These qualitative findings, which should be interpreted in relation to the sociodemographic and methodological context of the study, yield valuable learnings for research and practice. Healthcare students, healthcare educators, and lived experience experts discussed both the advantages and barriers associated with using VR to understand the experience of patients with MHCs. Furthermore, the recommendations put forward can inform the design, content, and delivery of VR-based interventions to reduce mental health stigma in healthcare education.

## Supplemental Material

sj-docx-1-dhj-10.1177_20552076231223801 - Supplemental material for ‘It's not everybody's snapshot. It's just an insight into that world’: A qualitative study of multiple perspectives towards understanding the mental health experience and addressing stigma in healthcare students through virtual reality Click here for additional data file. Supplemental material, sj-docx-1-dhj-10.1177_20552076231223801 for ‘It's not everybody's snapshot. It's just an insight into that world’: A qualitative study of multiple perspectives towards understanding the mental health experience and addressing stigma in healthcare students through virtual reality by Raul Szekely, Oliver Mason, David Frohlich and Elizabeth Barley in DIGITAL HEALTH

sj-docx-2-dhj-10.1177_20552076231223801 - Supplemental material for ‘It's not everybody's snapshot. It's just an insight into that world’: A qualitative study of multiple perspectives towards understanding the mental health experience and addressing stigma in healthcare students through virtual reality Click here for additional data file. Supplemental material, sj-docx-2-dhj-10.1177_20552076231223801 for ‘It's not everybody's snapshot. It's just an insight into that world’: A qualitative study of multiple perspectives towards understanding the mental health experience and addressing stigma in healthcare students through virtual reality by Raul Szekely, Oliver Mason, David Frohlich and Elizabeth Barley in DIGITAL HEALTH

sj-docx-3-dhj-10.1177_20552076231223801 - Supplemental material for ‘It's not everybody's snapshot. It's just an insight into that world’: A qualitative study of multiple perspectives towards understanding the mental health experience and addressing stigma in healthcare students through virtual reality Click here for additional data file. Supplemental material, sj-docx-3-dhj-10.1177_20552076231223801 for ‘It's not everybody's snapshot. It's just an insight into that world’: A qualitative study of multiple perspectives towards understanding the mental health experience and addressing stigma in healthcare students through virtual reality by Raul Szekely, Oliver Mason, David Frohlich and Elizabeth Barley in DIGITAL HEALTH

sj-docx-4-dhj-10.1177_20552076231223801 - Supplemental material for ‘It's not everybody's snapshot. It's just an insight into that world’: A qualitative study of multiple perspectives towards understanding the mental health experience and addressing stigma in healthcare students through virtual reality Click here for additional data file. Supplemental material, sj-docx-4-dhj-10.1177_20552076231223801 for ‘It's not everybody's snapshot. It's just an insight into that world’: A qualitative study of multiple perspectives towards understanding the mental health experience and addressing stigma in healthcare students through virtual reality by Raul Szekely, Oliver Mason, David Frohlich and Elizabeth Barley in DIGITAL HEALTH
