# Supplementary material for: ‘It's not everybody's snapshot. It's just an insight into that world’: A qualitative study of multiple perspectives towards understanding the mental health experience and addressing stigma in healthcare students through virtual reality
Source: Digit Health. 2024 Jan 4;10:20552076231223801. doi: 10.1177/20552076231223801 (PMC10768613; doi:10.1177/20552076231223801)
Supplement: sj-docx-1-dhj-10.1177_20552076231223801 - Supplemental material for ‘It's not everybody's snapshot. It's just an insight into that world’: A qualitative study of multiple perspectives towards understanding the mental health experience and addressing stigma in healthcare students through virtual real [file sj-docx-1-dhj-10.1177_20552076231223801.docx]

**Topic guide**

General

- Welcome, and thank you for your participation. My name is Raul and I am a PhD student at the University of Surrey.
- Background information and what is going to happen today – VR demonstration, focus group discussion.
- Confidentiality and ground rules.
- Is there something that you would like to ask before we start? Is there something that concerns you or is not very clear?
- Are you happy to continue?

Questions

In general, what are your thoughts on VR interventions that simulate the experiences of someone with a mental health condition?

- For example, an intervention whereby users hear intrusive, distressing voices.

Reflecting on your experience, do you feel there is a need for delivering VR interventions tackling mental health stigma among healthcare students?

- Why/why not?

Do you believe a VR intervention will or can change the way healthcare students view and act towards patients with mental health conditions?

- Why/why not?

What do you think VR interventions should include to improve healthcare students’ attitudes and behaviours towards patients with mental health conditions?

- What is important to consider? Think about the design process, content, delivery, etc.

Do you see any advantages or benefits for VR interventions compared to other stigma reduction interventions?

- Other interventions may be watching an educational video about mental health or interacting with someone with a mental health condition.

In your opinion, what are some of the barriers or challenges associated with the implementation of VR interventions in the education of healthcare students?

- You may consider ethical aspects, costs, acceptability and uptake, etc.

Before we finish, are there any other questions or comments you would like to raise?

Wrapping up

- Thank you once again for your very helpful contributions.
- Participation vouchers will be sent via email.
- Remind participants how their data is going to be handled and the possibility to withdraw.
- Ask participants to keep a copy of the information/debrief sheet and sources of support.
- Make sure participants leave the room/building safely.
