# Supplementary material for: ‘It's not everybody's snapshot. It's just an insight into that world’: A qualitative study of multiple perspectives towards understanding the mental health experience and addressing stigma in healthcare students through virtual reality
Source: Digit Health. 2024 Jan 4;10:20552076231223801. doi: 10.1177/20552076231223801 (PMC10768613; doi:10.1177/20552076231223801)
Supplement: sj-docx-4-dhj-10.1177_20552076231223801 - Supplemental material for ‘It's not everybody's snapshot. It's just an insight into that world’: A qualitative study of multiple perspectives towards understanding the mental health experience and addressing stigma in healthcare students through virtual real [file sj-docx-4-dhj-10.1177_20552076231223801.docx]

**Researcher reflexivity**

At the time of conducting this study, the lead researcher was a 25-year old male PhD student with an educational background in organisational psychology and work experience in healthcare-related research. He undertook training in qualitative research methods, including sensitive interviewing, and received guidance from an experienced and multidisciplinary research team with expertise in clinical psychology, interaction design and digital media, and nursing.

Having a non-clinical background, the lead researcher was aware of the potential limitations in his understanding of the complexities of healthcare education compared to those actively involved in the field. However, he anticipated that the healthcare educators and students taking part would recognise the value of the research study. The researcher did not assume that all healthcare professionals exhibited stigmatising attitudes towards patients with mental health conditions but was of the belief that even the most well-intentioned professionals may hold implicit biases and stereotypes that could influence patient interactions and attitudes towards mental health.

In addition, the lead researcher was optimistic about the potential effectiveness of VR as a stigma reduction tool for healthcare students and trainees. However, he was conscious that VR would not be a comprehensive solution for all mental health stigma-related issues. As such, the research aimed to contribute to a nuanced understanding of the role of VR in tackling mental health stigma by considering diverse perspectives and to inform the development of interventions that integrated VR with other approaches to reduce mental health stigma in healthcare education and practice.

Furthermore, in conducting this study, the lead researcher acknowledged the influence of personal experiences with mental health difficulties on his research perspective. While not identifying as a lived experience researcher in the strict sense, he recognised the systemic and structural barriers that individuals with severe mental health difficulties would often encounter, including within healthcare settings.

Lastly, the lead researcher ensured that he had no pre-existing personal relationships with any of the participants involved in the study.
